# Risk factors and incidence of short-term complications following open reduction and internal fixation of scapula fractures

**DOI:** 10.1007/s00590-024-04045-y

**Published:** 2024-07-22

**Authors:** Nicholas R. Kiritsis, Charles R. Reiter, James R. Satalich, Omar Protzuk, Conor N. O’Neill, Jennifer L. Vanderbeck

**Affiliations:** 1 Wake Forest University School of Medicine, 1 Medical Center Blvd, Winston-Salem, NC 27157 USA; 2 Virginia Commonwealth University School of Medicine, 1000 E Marshall St, Richmond, VA 23298 USA; 3 Department of Orthopaedic Surgery, Virginia Commonwealth University Medical Center, 1200 E Broad St, 9th Floor, Box 980153, Richmond, VA 23298 USA; 4 Department of Orthopaedic Surgery, Duke University Health System, 2301 Erwin Rd, Durham, NC 27710 USA

**Keywords:** Scapula, Scapula fracture, Glenoid, Acromion, Coracoid

## Abstract

**Purpose:**

To determine the short-term complication rates following open reduction and internal fixation of scapula fractures, factors affecting the development of adverse events, and complication rates based on the anatomic location of the fracture.

**Methods:**

Thirty-day complication rates for patients who underwent open reduction and internal fixation of the scapula were compared between glenoid, body, coracoid, and acromion fracture locations, as identified by International Classification of Disease codes. Possible adverse events included postoperative surgical site infection, renal insufficiency, intubation, pneumonia, deep vein thrombosis, pulmonary embolism, urinary tract infection, wound dehiscence, stroke, and blood transfusion.

**Results:**

A total of 251 scapula fractures were identified, with 161 having known fracture locations: 105 glenoid, 20 body, 9 coracoid, and 27 acromial fractures. The rate of any adverse event for all scapular fractures was 2.0%, with no significant difference between anatomic locations (*p* = 0.79). The overall rates of transfusion, surgical site infection, and return to OR were 0.4%, 0.8%, and 3.98%. Steroid use associated with a significantly increased risk of any adverse event (OR: 55.57, *p* = 0.038) and outpatient status demonstrated a protective effect on reoperation (OR: 0.11, *p* = 0.014). There were no significant differences in the rates between groups [transfusion (*p* = 0.91); surgical site infection (*p* = 0.17); reoperation (*p* = 0.85)].

**Conclusion:**

Complication rates within thirty days of ORIF for scapula fracture were low. Reoperation was the most common complication, followed by surgical site infection, wound dehiscence, stroke, transfusion, and pneumonia. Steroid use was a risk factor for developing any adverse event, and outpatient status was protective against reoperation. The 30-day complication profile of glenoid, body, coracoid, and acromial fractures was not significantly different. The low complication rates support the relative short-term safety of operative intervention with internal fixation.

**Level of Evidence:**

Level III.

## Introduction

Fractures of the scapula are relatively rare, comprising roughly 3–5% of all upper extremity fractures and 0.5% of all orthopedic fractures [1]. They most commonly occur in the setting of direct trauma to the shoulder, chest, or upper back. Due to protection offered by nearby osseous and soft tissue structures, a large amount of force is required to fracture the scapula. In the setting of a scapula fracture, patients will have associated injuries in 80–95% of cases which must be taken into consideration when planning treatment. Fractures can be classified based on anatomic location including the body, spine, neck, glenoid, coracoid, and acromion of the scapula, and the specific morphology may play a role in surgical decision-making and outcomes. Many scapula fracture types can be successfully treated with conservative methods such as brief immobilization and rehabilitation [2–4]. Nonunion rates are relatively low in both operative and nonoperative treatment of scapula fractures, and it is postulated that the scapula’s soft tissue envelope, rich blood supply, and multiple muscle attachments provide an optimal environment for fracture healing. Furthermore, patients can compensate for scapular deformity with a wide range of glenohumeral motion coupled with scapulothoracic motion in order to maintain native shoulder motion and strength [3, 4]. In recent years, however, there has been a shift in treatment paradigms, and no clear consensus on when to intervene surgically in these injuries exists to date.

Historically, there has been controversy regarding operative indications for scapula fractures. Greatly displaced or angulated fractures can alter rotator cuff tension and shoulder biomechanics, and cause weakness. Additionally, despite the robust fracture healing, scapular mal-unions and nonunions can severely impact shoulder function and cause chronic pain, impingement, scapulothoracic dyskinesis, and esthetic issues [5]. While relative indications for surgical fixation of the scapula have been suggested, consensus has yet to be determined. That being said, recent advances in procedural techniques have given surgeons more confidence in pursuing surgical fixation over conservative treatment. A review by Zlowodzki et al. found that in the early 2000s 80% of intra-articular glenoid fractures were treated surgically, compared to only 1% for isolated body fractures [3]. A more recent review saw nearly 57% (53/94) of body fractures receive operative management [6]. Surgical fixation provides superior anatomic alignment and restoration of shoulder joint biomechanics and function than conservative treatment [6]. Furthermore, in the setting of intra-articular fracture, open anatomic reduction and surgical fixation reduce the risk of acute instability and long-term degenerative joint disease compared to conservative treatment [7]. While surgical fixation is reliable and achieves positive results with good to excellent functional outcomes, it may carry a greater risk of complications compared to nonoperative treatment [3, 6]. It is vital to delineate a risk profile associated with scapular fracture surgical fixation as this may play a role in determining the need for operative intervention [8].

There are few studies evaluating the short-term complications associated with scapula fractures, with even fewer investigating perioperative risks following open reduction internal fixation. Understanding the perioperative risk associated with these procedures is important to appropriately counsel patients on the individual risks and benefits of pursuing surgery. The purpose of this study was to determine the short-term complication rates of surgical fixation of scapula fractures, factors affecting the development of adverse events, and complication rates based on anatomic location.

## Materials and methods

This is a retrospective cohort study of prospectively collected data as part of the American College of Surgeons National Quality Improvement Program (ACS-NSQIP) database. This registry contains demographics, comorbidities, and laboratory values with corresponding readmission and complication rates within thirty days of the indexed procedure. Patients are identified through Current Procedural Terminology (CPT) and International Classification of Diseases Ninth and Tenth Revision (ICD-9, ICD-10) codes [9]. NSQIP hospitals each employ trained nurse surgical clinical reviewers (SCRs) to oversee data collection adding an additional quality measure. All patients are monitored for thirty days postoperatively for any adverse events, readmissions, and reoperations. No outcome differences exist between institutions participating in the NSQIP with nonparticipants [10]. The ACS-NSQIP database is comprised of a network of hospitals which are required to employ SCRs to collect 274 variables from surgical procedures. The database implements several quality assurance measures, such as biweekly random internal audits, which have reported less than 1.8% inter-rater disagreement, and has been well established and reliable for study within orthopedic surgery [11–15].

Patients were identified based on codes for open treatment of scapular fracture with internal fixation (CPT 23585) from 2012 to 2021. Those with concomitant procedures during the operation were excluded. Limiting our study from 2012 to 2021 accounted for recent trends in scapula fixation methods available within the dataset while still gathering a large and representative sample size. Using International Classification of Disease (ICD) codes, cases were divided by their anatomic location within the scapula: glenoid, body, coracoid, or acromion. Patient demographics including age, gender, race, smoking status, body mass index (BMI), and American Society of Anesthesiologists (ASA) physical status classification score were collected along with complications data for each.

For the overall population and those with known fracture locations, 30-day incidence of postoperative complication was identified. For each patient, length of stay, nonhome discharge disposition, readmission rate, and 30-day complications were collected. Complications queried included: superficial and deep surgical site infections, pneumonia, postoperative reintubation or failure to wean from ventilator, deep vein thrombosis, pulmonary embolism, postoperative renal insufficiency or failure, urinary tract infection, wound dehiscence, stroke, and transfusion. Return to OR was defined as a return to the operating room for a major surgical procedure of any kind within 30 days of index operation. Length of stay was defined as the number of days from procedure to postoperative discharge. The primary outcome of this study was the overall rate of any adverse event and the above complications. Secondary outcomes were factors influencing the development of adverse events and differences in perioperative variables with respect to anatomic location of scapular fracture. Statistical analysis was performed using JMP software (Version 16.0, SAS Institute Inc., Cary, NC). Multivariable logistic regression with robust error variance was performed identify patient risk factors for AAE and unplanned hospital readmission. All demographic variables were included in the models as potential predictors. Statistical significance was set a priori at *p* < 0.05. Complications were compared between anatomic fracture location cohorts using an analysis of variance (ANOVA) test. Throughout these analyses, statistical significance was achieved with *p* < 0.05.

## Results

A total of 251 scapula fractures undergoing open reduction and internal fixation were identified for evaluation in our study. For all patients, the mean age was 49.5 ± 17.0 years, mean BMI was 29.7 ± 6.4, and mean ASA class was 2.0 ± 0.7 (Table 1). The anatomic fracture locations of 161 patients with known fracture locations were identified as follows: 105 glenoid, 20 body, 9 coracoid, and 27 acromial fractures.

**Table 1:** Demographic and comorbidity characteristics for patients undergoing scapula fracture repair by fracture location

	All patients	Glenoid	Body	Coracoid	Acromion
*n* (%)	251	105 (65.2%)	20 (12.4%)	9 (5.6%)	27 (16.8%)
Sex (male/female)	193 // 58	85 // 20	19 // 1	7 // 2	19 // 8
Percent male	76.9%	80.9%	95.0%	77.8%	70.4%
Age (mean ± SD)	49.5 ± 17.0	48.3 ± 13.9	43.1 ± 14.6	35.6 ± 8.7	54.7 ± 20.3
BMI (mean ± SD)	29.7 ± 6.4	30.9 ± 6.9	26.4 ± 4.4	27.9 ± 3.7	29.2 ± 6.7
Outpatient, *n* (%)	159 (63.3%)	65 (61.9%)	9 (45.0%)	8 (88.9%)	23 (85.2%)
ASA (mean ± SD)	2.0 ± 0.7	1.9 ± 0.7	1.8 ± 0.8	1.8 ± 0.4	2.2 ± 0.6
White, *n* (%)	171 (68.1%)	65 (61.9%)	15 (75.0%)	4 (44.4%)	22 (81.5%)
Black, *n* (%)	19 (7.6%)	7 (6.7%)	1 (5.0%)	2 (22.2%)	1 (3.7%)
Asian, *n* (%)	5 (2.0%)	1 (0.9%)	0 (0%)	0 (0%)	2 (7.4%)
Other race, *n* (%)	56 (22.3%)	32 (30.5%)	4 (20.0%)	3 (33.3%)	2 (7.4%)
Current smoker, *n* (%)	40 (15.9%)	16 (15.2%)	3 (15.0%)	3 (33.3%)	6 (22.2%)
Diabetes mellitus, *n* (%)	20 (8.0%)	8 (7.6%)	1 (5.0%)	0 (0%)	3 (11.1%)

251 patients were included in this analysis, with 161 having known fracture locations

Out of 105 patients with glenoid fractures, 80.9% were male. The average age was 48.3 ± 13.9 years, with a BMI of 30.9 ± 6.9 kg/m^2^. The body fracture group consisted of 20 patients, with a male predominance of 95.0%. The average age in this group was 43.1 ± 14.6 years, and the BMI was 26.4 ± 4.4 kg/m^2^. Coracoid fractures comprised 9 patients, of which 77.8% were male. Patients had an average age of 35.6 ± 8.7 years and a BMI of 27.9 ± 3.7 kg/m^2^. Acromion fractures were seen in 27 patients, with a 70.4% male predominance. The average age was 54.7 ± 20.3 years, with a BMI of 29.2 ± 6.7 kg/m^2^ (Table 1).

The overall rate of any adverse event was 2.0%. Surgical site infection was the most common complication (0.8%), followed by wound dehiscence (0.4%), stroke (0.4%), transfusion (0.4%), and pneumonia (0.4%). Unplanned return to the operating room was seen in 3.98% of patients and an unplanned readmission was necessary for 4 patients (1.59%), as given in Table 2.

**Table 2:** Incidence of adverse events for all fractures (*n* = 251)

	No.	Rate (%)
Any adverse event	5	2.0
Death	0	0.0
Wound dehiscence	1	0.4
Sepsis	0	0.0
Pulmonary embolism	0	0.0
Renal complication	0	0.0
Myocardial infarction	0	0.0
Cardiac arrest	0	0.0
Stroke	1	0.4
Transfusion	1	0.4
Deep vein thrombosis	0	0.0
Urinary tract infection	0	0.0
Pneumonia	1	0.4
Intubation issues	0	0.0
Surgical site infection	2	0.8
Return to the operating room	10	3.98
Readmission	4	1.59

Any adverse event included surgical site infection, pneumonia, postoperative reintubation or failure to wean from ventilator, deep vein thrombosis, pulmonary embolism, postoperative renal insufficiency or failure, urinary tract infection, wound dehiscence, stroke, and transfusion

Steroid use was the only risk factor associated with a significantly increased risk of any adverse event within 30 days of operation (OR: 55.57, *p* = 0.038). Outpatient status demonstrated a protective effect on reoperation (OR: 0.11, *p* = 0.014) and was the only risk factor associated with a significant relationship to reoperation. No other significant relationships were observed between the queried risk factors and odds of any adverse event or reoperation (Tables 3 and 4).

**Table 3:** Multivariate analysis of risk factors for any adverse event (AAE) after ORIF of scapula fracture, related to patient demographics and comorbidities

	OR coefficient	2.5% OR	97.5% OR	*p*
Age (1-year intervals)	1	0.93	1.07	0.999
BMI	0.8	0.58	1.11	0.178
Male sex	0.29	0.03	3.27	0.319
Operative time	0.98	0.96	1.01	0.207
Outpatient status	0.24	0.02	2.68	0.247
Dependent functional status	0	0	Inf	0.999
Current smoker	0	0	Inf	0.997
Dialysis	5.24 × 10^14^	0	Inf	0.999
Steroid use	55.57	1.26	2453.57	*0.038
Hypertension	0.29	0.01	13.40	0.525
Bleeding disorder	0	0	Inf	0.999
Diabetes	14.85	0.21	1058.50	0.215
COPD	0	0	Inf	0.999

Hypertension refers to hypertension requiring medication; dialysis refers to acute or chronic renal failure requiring dialysis within 2 weeks of indexed procedure

*BMI*, body mass index; *COPD*, chronic obstructive pulmonary disease

*Denotes statistical significance (*p* < 0.05)

**Table 4:** Multivariate analysis of risk factors for reoperation after ORIF of scapula fracture, related to patient demographics and comorbidities

	OR coefficient	2.5% OR	97.5% OR	*p*
Age (1-year intervals)	0.98	0.94	1.03	0.485
BMI	1.06	0.95	1.18	0.305
Male sex	0.86	0.15	5.08	0.87
Operative time	0.99	0.98	1.00	0.161
Outpatient status	0.11	0.02	0.64	*0.014
Dependent functional status	1.72 × 10^9^	0	Inf	0.998
Current smoker	0	0	Inf	0.994
Dialysis	0	0	Inf	0.999
Steroid use	1.24 × 10^9^	0	Inf	0.994
Hypertension	1.66	0.24	11.41	0.604
Bleeding disorder	0	0	Inf	0.999
Diabetes	0	0	Inf	0.994
COPD	0	0	Inf	0.997

Hypertension refers to hypertension requiring medication; dialysis refers to acute or chronic renal failure requiring dialysis within 2 weeks of indexed procedure

*BMI*, body mass index; *COPD*, chronic obstructive pulmonary disease

*Denotes statistical significance (*p* < 0.05)

There was no significant difference in overall complication rates based on anatomic location (*p* = 0.79). The acromion cohort experienced the highest rate of adverse events at 3.7%, followed by glenoid fractures (0.95%). Scapular body and coracoid fractures did not demonstrate an adverse event. Unplanned reoperation was highest in the acromion cohort (7.4%), followed by scapular body (5%), glenoid (3.8%), and coracoid (0%). No rate of individual adverse events was significantly different between the fracture locations [infection (*p* = 0.17); transfusion (*p* = 0.91); reoperation (*p* = 0.85)]. Length of stay was different between groups (*p* = 0.01) (Table 5).

**Table 5:** Incidence of adverse events by fracture location

	Glenoid	Body	Coracoid	Acromion	*p*
Any adverse event, *n* (%)	1 (0.95%)	0 (0%)	0 (0%)	1 (3.7%)	0.79
Surgical site infection, *n* (%)	0 (0%)	0 (0%)	0 (0%)	1 (3.7%)	0.17
Blood transfusion, *n* (%)	1 (1.0%)	0 (0%)	0 (0%)	0 (0%)	0.91
Return to the OR, *n* (%)	4 (3.8%)	1 (5.0%)	0 (0%)	2 (7.4%)	0.85
Length of stay, days (mean ± SD)	0.97 ± 1.6	1.80 ± 2.5	0.22 ± 0.4	0.37 ± 0.6	*0.01

Any adverse event included surgical site infection, pneumonia, postoperative reintubation or failure to wean from ventilator, deep vein thrombosis, pulmonary embolism, postoperative renal insufficiency or failure, urinary tract infection, wound dehiscence, stroke, and transfusion

*Denotes statistical significance (*p* < 0.05)

## Discussion

There is a paucity of literature investigating short-term complication rates associated with surgical fixation of scapula fractures, yet understanding perioperative complications of these procedures is important for patient counseling and informed consent. This retrospective cohort study identified patients from the ACS-NSQIP database who underwent open treatment of scapula fracture including internal fixation from 2012 to 2021 and identified the overall 30-day incidence of postoperative complications and for each anatomic fracture location. A total of 251 scapula fractures were identified, with 161 having known fracture locations: 105 glenoid, 20 body, 9 coracoid, and 27 acromial fractures. The rate of any adverse events for all scapular fractures was 2.0%, with a 3.98% rate of return to the operating room, 0.8% rate of surgical site infection, 0.4% rate of dehiscence, 0.4% rate of stroke, 0.4% rate of transfusion, and 0.4% rate of pneumonia. Steroid use was a risk factor for developing any adverse event, and outpatient status was protective against reoperation. There were no significant differences in complication rates between anatomic locations, though there was a difference in average length of stay.

Even with involvement of varying anatomic structures, the complication profile of repairing glenoid, body, coracoid, and acromial fractures was not statistically different. These results support several prior studies that demonstrate low rates of adverse events, including infection and return to the operating room. Both Bauer et al. and Cole et al. found a short-term complication rate of 0% following open reduction internal fixation of scapula fractures in their cohorts of 20 and 6 patients, respectively [16, 17]. Similarly, Vidović et al. saw no postoperative infections in their study of 14 patients [18]. While these were smaller studies, our results support these findings with a larger sample. Prior systematic reviews have also found complication rates similar to our findings. In a review of 212 cases, 4.2% of patients experienced an infection, though the timeframe was not specified [19]. In a cohort of 20 patients with surgically repaired scapular body fractures, Rollo et al. found a 5% rate of surgical site infection and reoperation, compared to 0% and 5.0% for scapular body fractures in our analysis [20]. The discrepancy may in part be due to their longer follow-up time of at least 12 months.

The Scapula Institute’s current recommendations for surgical management take into consideration the patient’s age, prior function, fracture characteristics including articular displacement, percentage of articular involvement, and glenopolar angle among other factors [5]. In the instance of a floating shoulder or open fracture, prompt operative management is warranted. Our findings suggest that the fracture location should not impact the perioperative and short-term postoperative risk assessment for ORIF of scapula fractures. Imaging can further assist in determining recommendations for operative fixation of scapula fractures. These include more relative indications including articular step-off > 5 mm, glenohumeral instability, anterior rim fracture, posterior rim fracture, angular deformity > 40°, lateral border offset > 15 mm with angular deformity > 35°, lateral border offset > 20 mm, and glenopolar angle < 22° [8, 21].

Surgical approaches for repairing scapula fractures can vary based on the location and involved anatomic structures. The most commonly used approach for scapula fixation is the posterior (Judet) approach, which adequately exposes the inferior glenoid and body for improved visualization [22]. In a review of 166 cases, nearly 80% of fractures were fixed with a posterior approach, while only 18.1% used an anterior approach and 3% used a combination anterior and posterior [19]. Posterior approaches are recommended except in the case of anterior glenoid fractures, superior glenoid fractures involving the coracoid, or isolated process fractures [19]. In our analysis, surgical approach was not reported for each patient, which may have impacted the observed complication rates.

There are several limitations to our study including the retrospective nature of the data collection. We did not perform a pre-investigation power analysis, so our study may be underpowered, and therefore, all nonsignificant findings should be interpreted with caution. Fracture pattern or type or number of implants used for surgical fixation was not considered, which may introduce bias. There was no specification of the surgical approach used for each patient, which may impact the observed complication rates. The NSQIP database does not document the experience of the primary surgeon, which likely affects complication rates. We did not include a control group in our study, but future research should include comparing rates of adverse events between nonoperative and operative groups. Finally, we did not investigate how union rates, functional measures, and patient-reported outcome scores relate to adverse events.

## Conclusion

Patients who underwent open reduction and internal fixation for scapula fractures had low complication rates in the short-term postoperative period (≤ 30 days). Reoperation was the most common complication, followed by surgical site infection, wound dehiscence, stroke, transfusion, and pneumonia. Steroid use was a risk factor for developing any adverse event, and outpatient status was associated with a reduced risk of reoperation. The complication profile of the glenoid, body, coracoid, and acromial fractures was not significantly different. These findings support the relative safety of operative intervention with internal fixation of the scapula in the short term. These data can improve patient counseling and decision-making when considering surgical treatment for scapula fractures.
